# MITOCHONDRIAL SYSTEM BIOLOGY AS A WINDOW INTO DISEASES OF AGING

**DOI:** 10.1093/geroni/igz038.2047

**Published:** 2019-11-08

**Authors:** Pinchas Cohen

**Affiliations:** Leonard Davis School of Gerontology, Los Angeles, California, United States

## Abstract

We identified multiple open-reading-frames (ORFs) within the mitochondrial genome. These ORFs encode putative peptides that we call Mitochondrial-Derived-Peptides (MDPs) which represent a sub-class of a growing group of novel micro-peptides (from both mtDNA and nuclear chromosomes) that serve as signals related to cell and organismal protection and energy expenditure. We described multiple peptides including humanin, SHLPs, and MOTS-c. Exploring mtDNA methylation patterns as well as mito-transcriptomics demonstrated changes in specific ORFs/MDPs in certain diseases. We developed a modified GWAS bioinformatic technique (MiWAS) that identifies SNPs within MDPs that associate with diseases of aging. MOTS-c, and MENTSH, novel anti-obesity/diabetes MDPs, harbors mutation in Asians and Native-Americans, associated with diabetes risk. Thus, MDPs are expressed in an ethno-specific fashion and may contribute to health disparities in a manner related to relevant mitochondrial DNA SNPs. In summary, MDPs are a new class of mitochondrial-hormones that have diagnostic and therapeutic potential in human disease.

